# Brief Subthreshold Events Can Act as Hebbian Signals for Long-Term Plasticity

**DOI:** 10.1371/journal.pone.0006557

**Published:** 2009-08-07

**Authors:** Elodie Fino, Jean-Michel Deniau, Laurent Venance

**Affiliations:** Dynamics and Pathophysiology of Neuronal Networks, INSERM U-667, Collège de France, University Pierre et Marie Curie, Paris, France; INSERM U862, France

## Abstract

**Background:**

Action potentials are thought to be determinant for the induction of long-term synaptic plasticity, the cellular basis of learning and memory. However, neuronal activity does not lead systematically to an action potential but also, in many cases, to synaptic depolarizing subthreshold events. This is particularly exemplified in corticostriatal information processing. Indeed, the striatum integrates information from the whole cerebral cortex and, due to the membrane properties of striatal medium spiny neurons, cortical inputs do not systematically trigger an action potential but a wide range of subthreshold postsynaptic depolarizations. Accordingly, we have addressed the following question: does a brief subthreshold event act as a Hebbian signal and induce long-term synaptic efficacy changes?

**Methodology/Principal Findings:**

Here, using perforated patch-clamp recordings on rat brain corticostriatal slices, we demonstrate, that brief (30 ms) subthreshold depolarizing events in quasi-coincidence with presynaptic activity can act as Hebbian signals and are sufficient to induce long-term synaptic plasticity at corticostriatal synapses. This “subthreshold-depolarization dependent plasticity” (SDDP) induces strong, significant and bidirectional long-term synaptic efficacy changes at a very high occurrence (81%) for time intervals between pre- and postsynaptic stimulations (Δt) of −110<Δt<+110 ms. Such subthreshold depolarizations are able to induce robust long-term depression (cannabinoid type-1 receptor-activation dependent) as well as long-term potentiation (NMDA receptor-activation dependent).

**Conclusion/Significance:**

Our data show the existence of a robust, reliable and timing-dependent bidirectional long-term plasticity induced by brief subthreshold events paired with presynaptic activity. The existence of a subthreshold-depolarization dependent plasticity extends considerably, beyond the action potential, the neuron's capabilities to express long-term synaptic efficacy changes.

## Introduction

Learning and memory are thought to involve long-term synaptic efficacy changes [1]–[3]. In the current conception of activity-dependent plasticity, the action potential constitutes the physiologically pertinent coding event determinant for the induction of long-term synaptic plasticity. Accordingly, action potentials are classically referred as Hebbian signals. The key role of the action potential is exemplified by spike-timing dependent plasticity (STDP), in which the timing between pre- and postsynaptic action potentials rules the induction of long-term synaptic efficacy changes [4]–[8]. However, synaptic transmission does not necessarily lead to the triggering of a postsynaptic action potential. Does that mean that brief subthreshold depolarizing signals cannot induce long-term synaptic efficacy changes and are consequently lost for such processes? We have tested this hypothesis at corticostriatal synapses where numerous subthreshold events occur during cerebral cortex activity. Some studies indicate that the postsynaptic action potential would not be the only postsynaptic depolarizing event necessary for the induction of long-term synaptic plasticity [9]–[12]. However, these studies applied changes of holding membrane potential of the postsynaptic neuron for long duration and have reported the exclusive induction of either LTP or LTD, but never a bidirectional plasticity. We have tested here the effect of brief postsynaptic subthreshold depolarizations on the induction of long-term synaptic plasticity and therefore their ability to act as Hebbian signals.

The corticostriatal pathway provides the first step of cortical information processing in basal ganglia, an ensemble of interconnected sub-cortical nuclei involved in learning of contextual cognitive-motor sequences related to environmental stimuli [13]–[18]. The striatum, the main input nucleus of basal ganglia, receives glutamatergic inputs from the whole cerebral cortex. In turn, it relays the integrated cortical information towards the basal ganglia output nuclei through which it operates a selected activation of behavioral effectors. The medium-sized spiny neurons (MSNs) represent the main neuronal striatal population and act as detectors and integrators of distributed patterns of cortical activity [19], [20]. Due to their membrane properties, MSNs are silent at rest and need strong and correlated inputs to discharge [21]–[23]. Consequently, cortical inputs do not systematically lead to an action potential but to a wide range of postsynaptic depolarizations, which mostly remain subthreshold [24]–[26]. Therefore, we have addressed the following question: can brief subthreshold depolarizing events act as Hebbian signal? Accordingly, we have investigated, using perforated patch-clamp techniques, if brief subthreshold signals could be involved in the induction of long-term corticostriatal synaptic plasticity.

## Results

### Corticostriatal monosynaptic transmission

**Figure 1:**
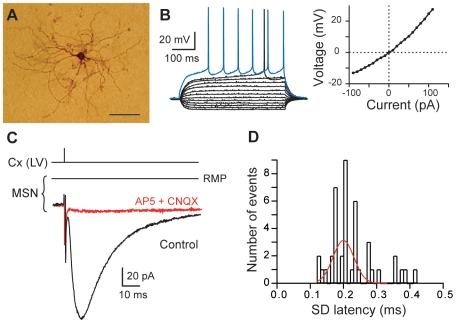
MSN characterization and corticostriatal monosynaptic transmission. (A) High magnification of a MSN injected with biocytin (scale bar, 100 µm). (B) MSN membrane properties and spiking pattern: a hyperpolarized RMP, an inward rectification (illustrated in the steady-state I-V relationship) and a long depolarizing ramp to the action potential threshold leading to a delayed spike discharge. Raw traces show voltage responses to 500 ms current pulses from −90 pA to 110 pA with 20 pA steps and to +40 pA (blue trace) above action potential threshold. (C) Cortically-evoked MSN EPSCs (averages of 7 traces) in control and with CNQX (10 µM) and AP5 (50 µM). (D) Distribution of latency SD was centered on 0.20 ms and fitted by a Gaussian function. These values of SD latency indicate a monosynaptic corticostriatal transmission because inferior to 0.5 ms. Cx (LV), cortical layer V.

We have used horizontal rat brain slices in which connections between pyramidal cells of cerebral cortex and MSNs were preserved [27]–[29]. We have performed electrical stimulation in layer V of the somatosensory cortex while recording MSNs by perforated patch-clamp in the functionally related region, the dorsal striatum. MSNs were identified morphologically (medium-sized soma with highly branched spiny dendrites; Fig. 1A) and electrophysiologically: a hyperpolarized resting membrane potential (RMP; −73.4±0.6 mV, n = 75), an inward rectification of I-V relationship, a long depolarizing ramp to spike threshold, a long delay to first spike (384±6 ms, for 500 ms depolarizing pulses) evoked at rheobase (Fig. 1B). Stimulations of cortical afferents evoked glutamatergic excitatory postsynaptic currents (EPSCs) (inhibited by CNQX 10 µM and AP5 50 µM, n = 5) in MSNs (Fig. 1C) with a success rate of 97% (n = 72). Once corticostriatal transmission occurred, no failure was observed, indicating a reliable and efficient transmission. Cortically-evoked EPSCs displayed latencies with an average value of 2.32±0.03 ms, (n = 43 MSNs). Transmission was monosynaptic since the standard deviations of latencies were inferior to 0.5 ms (0.24±0.02 ms, n = 43 MSNs) and displayed a very narrow Gaussian distribution (Fig. 1D).

### Subthreshold-depolarization dependent plasticity (SDDP)

**Figure 2:**
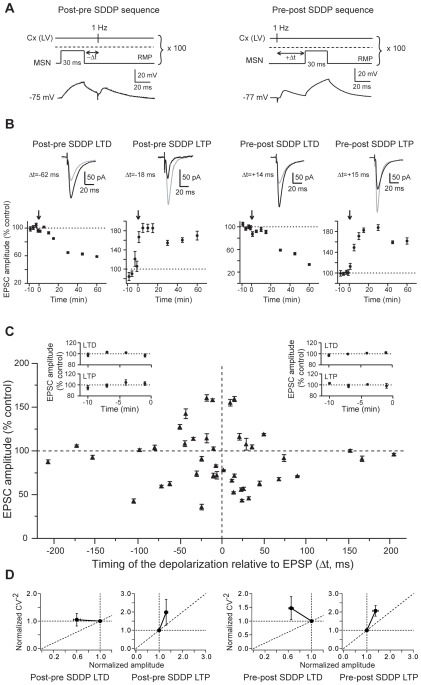
Postsynaptic subthreshold depolarizations paired with presynaptic stimulations induce long-term synaptic plasticity. (A) SDDP protocols and the corresponding raw traces of the postsynaptic MSN recordings. Brief subthreshold depolarizations were evoked in the MSN just before (post-pre SDDP sequences) or after (pre-post SDDP sequences) cortical stimulation (100 paired stimulations at 1 Hz). The dashed line indicates the action potential threshold. (B) Representative experiments of SDDP for the four observed cases. Long-term synaptic efficacy changes (illustrated by EPSCs evoked in control, black, and 60 minutes after cellular conditioning protocol, grey) were −37.5±1.4% for post-pre sdLTD (Δt = −62 ms), +60.5±4.9% for post-pre sdLTP (Δt = −18 ms), −47.5±2.0% for pre-post sdLTD (Δt = +14 ms) and +59.3±2.7% for pre-post sdLTP (Δt = +15 ms) (arrows indicate the cellular conditioning protocols). (C) SDDP protocols induce bidirectional long-term synaptic plasticity (each 43 MSN is indicated by black triangle, mean±SEM measured 60 minutes after cellular conditioning protocol). Post-pre protocols induced sdLTD or sdLTP for −110<Δt<0 ms (n = 19). Pre-post protocols induced mainly sdLTD for 0<Δt<+110 ms (n = 18). No long-term plasticity occurred for Δt beyond +/−110 ms (n = 6). Inserts: Averages of normalized EPSC amplitudes recorded in control before SDDP protocols inducing post-pre sdLTD (n = 7), post-pre sdLTP (n = 8), pre-post sdLTD (n = 12) and pre-post sdLTP (n = 4). Control EPSCs amplitudes were recorded for 10 minutes (minus sign indicates time before the SDDP protocol) and displayed no significant variations. (D) Normalized CV^−2^ were plotted as a function of the normalized EPSC amplitude to determine the loci of the SDDP. The four graphs illustrate the CV^−2^ plots (from left to right) for post-pre sdLTD, post-pre sdLTP, pre-post sdLTD and pre-post sdLTP. Mean variance analysis suggest that post-pre and pre-post sdLTD has mainly a postsynaptic origin and, post-pre and pre-post sdLTP is mainly underlain by presynaptic mechanisms.

**Figure 3:**
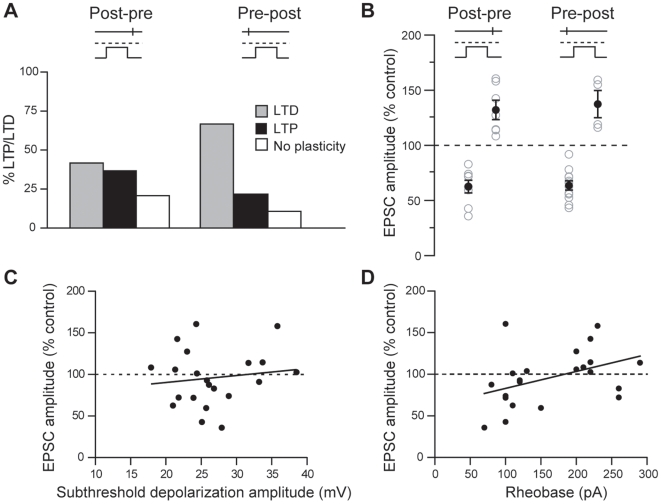
Characterization of SDDP. (A) Occurrences of sdLTD and sdLTP. Post-pre sequences (for −110<Δt<0 ms) induced sdLTD and sdLTP with success rates of 42% and 37%, respectively, and pre-post sequences (for 0<Δt<+110 ms) induced sdLTD (67%) and sdLTP (22%). (B) Magnitudes of long-term synaptic efficacy changes induced by post-pre and pre-post SDDP sequences: −37.4±5.7% and +32.1±8.9% for post-pre sdLTD and sdLTP, and −36.4±4.7% and +37.4±12.3% for pre-post sdLTD and sdLTP. (C) No correlation was found when the magnitudes of long-term plasticity induced by post-pre sequences were plotted against the amplitude of post-synaptic subthreshold depolarizations evoked during SDDP protocol (r = 0.147, p>0.05). (D) Rheobase and long-term synaptic plasticity induced by post-pre sequences in MSNs were significantly correlated (r = 0.417, p<0.05).

We tested whether postsynaptic brief subthreshold signals, in quasi-coincidence with corticostriatal presynaptic activity, could induce long-term synaptic efficacy changes in MSNs. We chose to evoke a brief postsynaptic subthreshold depolarizations (30 ms) to mimic corticostriatal subthreshold summation of EPSPs induced by cortical or thalamic activity (as observed in *in vivo* studies [24]–[26]) paired with a presynaptic cortical stimulation. Namely, a brief-duration (30 ms) subthreshold depolarization was induced in a single MSN a few milliseconds before (post-pre sequence) or after (pre-post sequence) a cortical afferent stimulation (100 paired stimulations at 1 Hz) (Fig. 2A). The amplitudes of the evoked postsynaptic subthreshold depolarizations (27.7±1.0 mV, n = 43 MSNs) were in accord with the subthreshold membrane potential transitions observed *in vivo* in MSNs [24]–[26]. Strikingly, brief subthreshold depolarizations paired with presynaptic activation were able to induce reliable and robust long-term synaptic plasticity (Fig. 2). Accordingly, we have named these long-term synaptic efficacy changes “subthreshold-depolarization dependent plasticity” (SDDP). Strong and significant SDDP were observed at a very high occurrence (81%, n = 43) indicating that brief subthreshold depolarizations were very effective in inducing long-term synaptic plasticity. Such plasticity was bidirectional, since post-pre and pre-post subthreshold depolarizations sequences induced subthreshold-depolarization long-term depression (sdLTD) or subthreshold-depolarization long-term potentiation (sdLTP) (Fig. 2 and 3). Nevertheless, depending on the applied SDDP sequences (post-pre versus pre-post), the occurrence of sdLTD and sdLTP was different.

For post-pre SDDP sequences, bidirectional long-term plasticities (sdLTD or sdLTP) occurred with a success rate of 79% for time intervals between pre- and postsynaptic stimulations (Δt) of −100<Δt<0 ms (n = 19) (Fig. 2C). Post-pre sdLTD and sdLTP were induced with a similar occurrence. Post-pre sdLTD and sdLTP are illustrated by representative experiments in Figure 2B. No long-term plasticity was observed for Δt beyond −110 ms (n = 3). For pre-post SDDP sequences, long-term synaptic plasticities occurred with a particularly high success rate of 89% for 0<Δt<+110 ms (n = 18). Representative experiments illustrate sdLTD and sdLTP induced by pre-post sequences (Fig. 2B). Interestingly, pre-post sequences induced a large majority of sdLTD (n = 12) compared to the induction of sdLTP (n = 4) (Fig. 2C and Fig. 3A). No long-term plasticity was observed for Δt>+110 ms (n = 3) (Fig. 2C). Mean variance analysis of the cortically-evoked synaptic events indicated that post-pre and pre-post sdLTD arose mainly from the postsynaptic element and post-pre and pre-post sdLTP is mainly underlain by presynaptic mechanisms (Fig. 2D).

### Characteristics of corticostriatal SDDP

Considering post-pre and pre-post sequences together (n = 37), during the time interval −110<Δt<+110 ms for which long-term plasticities were observed, sdLTD was induced twice as often (54%) as sdLTP (30%). Interestingly, depending on the Δt of post-pre and pre-post SDDP protocols, different proportions of sdLTD and sdLTP were observed (Fig. 3A). sdLTD/sdLTP occurrence ratios were 1.3 for post-pre (n = 18) and 3.3 for pre-post (n = 17) protocols (Fig. 3A). For intervals between -110<Δt<−50 and +50<Δt<+110 ms only sdLTD was induced and both sdLTD and sdLTP were induced for −50<Δt<+50 ms (Fig. 2C). Post-pre −50<Δt<0 ms sequences induced sdLTD or sdLTP, with a majority of sdLTP (sdLTD/sdLTP occurrence ratio was 0.7, n = 14) while pre-post 0<Δt<+50 ms sequences induced mainly sdLTD (sdLTD/sdLTP occurrence ratio was 2.3, n = 15). Importantly, the sequence order of pre- and postsynaptic events had no significant incidence on the magnitude of sdLTD or sdLTP (Fig. 3B). Considering the post-pre and the pre-post sequences respectively, the magnitudes of depression of EPSC amplitude observed for sdLTD were −37.4±5.7% (n = 8) and −36.4±4.7% (n = 12) and the magnitudes of potentiation observed for sdLTP were +32.1±8.9% (n = 7) and +37.4±12.3% (n = 4). These mean values were significantly different from the baseline recorded in control (p<0.01) (see averaged baseline data in Fig. 2C, inserts). We observed that pre-post sequences induced mainly sdLTD whereas post-pre sequences could induce sdLTP or sdLTD. Therefore, we investigated if occurrence and magnitude of post-pre SDDP could be predicted by parameters related to the induction protocol (evoked subthreshold depolarization amplitude), synaptic transmission (EPSC rise time, latency and amplitude before cellular conditioning) or neuronal membrane properties (RMP, input resistance and rheobase). We did not observe any significant correlation between subthreshold depolarization amplitudes and the magnitude of SDDP-evoked long-term plasticities, although a wide range of postsynaptic depolarization amplitudes was evoked (from 17.9 to 38.5 mV) (Fig. 3C). No significant correlation was observed between EPSC characteristics (rise time, latency or amplitude) or neuronal membrane properties (RMP and input resistance) and long-term synaptic efficacy changes (Fig. S1). Strikingly, rheobase was found to be a key component for the orientation of plasticities induced by a post-pre sequence (Fig. 3D). Indeed, a significant (p<0.05) correlation was observed between rheobase and the induced plasticity. MSNs displaying low rheobase values preferentially developed sdLTD whereas those with highest rheobases were associated with induction of sdLTP. Accordingly, excitability of MSNs appears to be determinant in the orientation of post-pre SDDP.

### SDDP needs a correlated timing between pre- and postsynaptic stimulations

**Figure 4:**
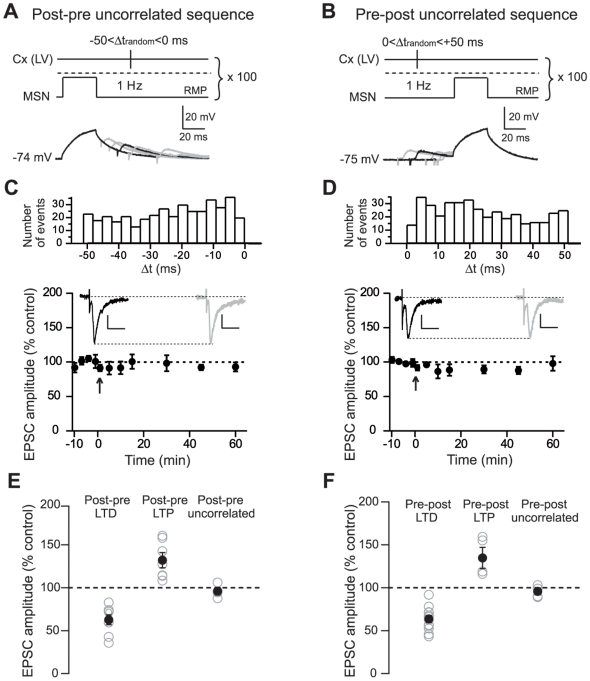
Uncorrelated random paired stimulations failed to induce significant long-term synaptic plasticity. (A–B) Uncorrelated post-pre (A) and pre-post (B) SDDP protocols consisted in varying randomly postsynaptic subthreshold depolarizations and presynaptic stimulations delays for each sweep of the pairing period Δt_random_ (range of random delays: −50 to 0 ms for post-pre sequences and 0 to +50 ms for pre-post sequences). Below: examples of five consecutive postsynaptic MSN recordings (superimposed raw traces) during post-pre (A) and pre-post (B) random pairings. (C–D) Mean effect of pairing with random delays for post-pre (n = 4 MSNs) (C) and for pre-post (n = 4 MSNs) (D) sequences. An absence of long-term synaptic efficacy changes was observed in both groups (post-pre and pre-post random pairings). Insets: EPSCs evoked before (black traces) or 60 minutes after the uncorrelated sequences (grey traces). Scale bars are 50 pA and 20 ms. Arrows indicate the cellular conditioning protocol. Upper panels show the distribution of random pairing delays across all cells for uncorrelated post-pre (−50<Δt_random_<0 ms) (E) and pre-post (0<Δt_random_<+50 ms) (F) SDDP sequences. (E–F) Long-term synaptic efficacy changes are illustrated for post-pre (E) and pre-post (F) correlated and uncorrelated paired SDDP. Open grey circles represent individual experiments and black circles represent average values. Post-pre (−50<Δt_random_<0 ms) or pre-post (0<Δt_random_<+50 ms) uncorrelated paired stimulations did not induce significant long-term plasticity in contrast to correlated pairings.

It has been demonstrated than STDP needs a correlated timing between pre- and postsynaptic stimulations [10]. Indeed, in case of random Δt pairing, STDP failed to induce long-term synaptic efficacy changes. When compared to an action potential, a 30 ms subthreshold depolarization is much wider. It is thus expected that SDDP coding should be temporally rather imprecise. We have tested this hypothesis with uncorrelated pre- and postsynaptic paired stimulations. Namely, the strength of the time-dependency of the SDDP was estimated by pairing EPSPs and subthreshold depolarizations at delays that varied randomly for each sweep of the pairing period (Fig. 4). We have generated random Δt for two different time windows: −50<Δt_random_<0 ms and 0<Δt_random_<+50 ms (Fig. 4A and B). Uncorrelated random sequences did not induce any significant plasticity as illustrated by the mean values of the synaptic efficacy changes obtained after uncorrelated post-pre sequences (n = 4) (Fig. 4C) or uncorrelated pre-post sequences (n = 4) (Fig. 4D). When delays between EPSPs and subthreshold depolarizations were varied randomly for −50<Δt<0 ms, no significant long-term synaptic efficacy changes was observed (−4.0±4.4%, n = 4; Fig. 4E). Similarly, a lack of significant plasticity (−4.2±4.0%, n = 4; Fig. 4F) was observed when pairing delays for 0<Δt_random_<+50. These experiments show that synapses that generate randomly paired EPSPs with postsynaptic subthreshold depolarizations in a time window of 50 ms become unable to display long-term efficacy changes. In conclusion, the occurrence of SDDP requires a precise timing between pre- and postsynaptic activities.

### sdLTD is CB1 receptor-activation dependent and sdLTP NMDA receptor-activation dependent

**Figure 5:**
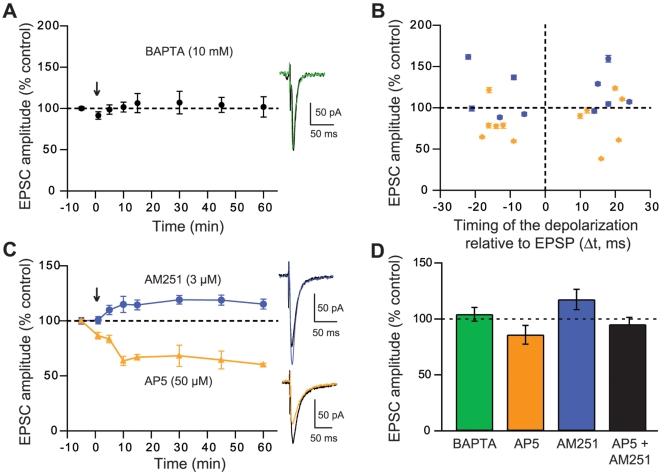
Pharmacological characterization of SDDP. (A) sdLTD and sdLTP are Ca^2+^-dependent. Mean effects of BAPTA for −50<Δt<+50 ms post-pre and pre-post SDDP sequences. When MSNs were loaded with BAPTA (10 mM, in whole-cell pipettes), SDDP protocols did not induce any long-term synaptic efficacy changes (+4.2±9.1%, n = 6, −50<Δt<+50 ms), indicating that the SDDP is Ca^2+^-dependent. The absence of synaptic efficacy changes is also illustrated by representative EPSCs evoked before (black traces) or 60 minutes after SDDP sequences (green trace). (B) Pharmacology of SDDP was explored for −30<Δt<+30 ms (each symbol represent one MSN, mean±SEM measured 60 minutes after cellular conditioning protocol). AP5 treatment (orange diamonds, n = 10) induces mainly sdLTD for post-pre and pre-post sequences. Treatment with AM251 (purple dots, n = 8) leads to sdLTP or absence of plasticity. (C) Representative experiments illustrate the effect of treatments with AM251 or AP5. In presence of AP5 (orange trace), a sdLTD is observed (−35.6±8.1%) and with AM251 treatment (purple trace), a sdLTP is observed (+18.8±4.6%). Representative EPSCs evoked before (black traces) or 60 minutes after SDDP sequences (orange or purple traces) also illustrate the long-term plasticity observed in presence of AP5 (sdLTD) or in presence of AM251 (sdLTP). (D) Histograms of long-term synaptic efficacy changes in BAPTA (green bars, n = 6), AP5 (orange bars, n = 12), AM251 (purple bars, n = 10) and with co-administration of AP5 and AM251 (black bars, n = 4): synaptic plasticity was Ca^2+^-dependent, sdLTD was CB1 receptor-activation dependent and sdLTP was NMDA receptor-activation dependent. Postsynaptic depolarizations evoked during pharmacological experiments were not significantly different than those induced for post-pre and pre-post SDDP sequences (27.4±1.1 mV *versus* 27.9±0.9 mV, respectively).

Ca^2+^ fluxes are commonly involved in synaptic plasticity. Accordingly, we tested the role of Ca^2+^ in the induction of SDDP with loading MSN of BAPTA (10 mM), a high affinity Ca^2+^ chelator, through whole-cell pipettes. Intracellular BAPTA loading prevented the induction of significant long-term synaptic efficacy changes (+4.1±6.1%, n = 6) by SDDP sequences (−50<Δt<+50 ms) (Fig. 5A and D), indicating that sdLTP and sdLTD are Ca^2+^-dependent.

We then explored which receptors were involved in the induction of SDDP. SDDP pharmacology was investigated for −30<Δt<+30 ms, an interval corresponding to the maxima of induction rate and magnitude of both sdLTD and sdLTP (Fig. 2C). With AP5 (a NMDA receptor antagonist, 50 µM) treatment (n = 12), we observed the occurrence of either sdLTD or an absence of plasticity, while no sdLTP was induced anymore (Fig. 5B). A representative experiment illustrates the induction of a sdLTD in presence of AP5 (Fig. 5C). With AP5 treatment, the average value of EPSC amplitude changes was −14.1±8.2% (n = 12) (Fig. 5D). Conversely, with AM251 (a cannabinoid type-1 receptor selective antagonist, 3 µM) bath application (n = 10), we observed exclusively the occurrence of sdLTP or an absence of plasticity, while no sdLTD was induced (Fig. 5B), as illustrated by the representative experiment (Fig. 5C). The average value of EPSC amplitude changes in presence of AM251 was +17.4±9.1% (n = 10) (Fig. 5D). Similar results were obtained when post-pre and pre-post SDDP protocols were considered separately (Fig. S2). These findings are consistent with the existence of independent pathways for sdLTP and sdLTD. In addition, the co-administration of AP5 and AM251 preclude the induction of long-term synaptic efficacy changes (95.0±6.4%, n = 4), either after post-pre or pre-post sequences (Fig. 5D).In conclusion, AP5 prevents the induction of sdLTP whereas AM251 prevents the induction of sdLTD indicating that sdLTD is CB1 receptor-activation dependent and sdLTP NMDA receptor-activation dependent.

### Is MSN firing a critical event for corticostriatal plasticity? Comparison of corticostriatal SDDP and STDP

**Figure 6:**
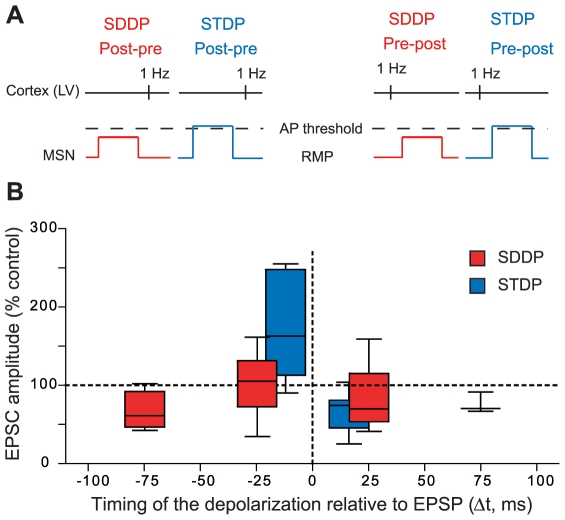
Comparison of the occurrence, orientation, magnitude and temporal extent of SDDP and STDP. (A) Schematic representations of post-pre and pre-post SDDP and STDP protocols (the two protocols differ by the presence or not of a postsynaptic action potential). (B) Long-term synaptic efficacy changes evoked by SDDP and STDP protocols illustrated with Box and Whiskers plots. When compared to SDDP, STDP changes were strictly orientated. SDDP changes were inducible in wider time windows than STDP (+/−110 *vs.* +/−30 ms). sdLTP were induced in a time window 1.7 fold wider than those of STDP LTP while the time window for sdLTD induction was 3.3 fold wider when compared to STDP LTD. Data concerning STDP experiments are taken partly from a previous study (Fino et al., 2005), with the addition of new experiments performed in perforated patch-clamp (n = 6).

We had previously characterized STDP at corticostriatal synapses [28]. Here, we performed additional experiments in perforated patch-clamp and confirmed that STDP was strictly orientated: post-pre STDP protocol induced LTP (78%, n = 18), while pre-post STDP protocol induced LTD (85%, n = 13) (Fig. 6B). Consequently, the direction of corticostriatal MSN STDP was reversed compared to that described so far in mammals [4]–[7].

The SDDP protocol applied in the present study uses a similar cortical afferent volley but differs from the STDP in the lack of occurrence of postsynaptic action potential (Fig. 6A). Remarkably, despite the lack of the postsynaptic action potential, SDDP protocols induced long-term synaptic efficacy changes with similar failure rate (∼20%) than STDP protocol. However, it should be noted that, even if magnitude averages concerning LTD and sdLTD are not significantly different, post-pre STDP protocols have the capability to induce LTP with a significantly higher magnitude than sdLTP induced by SDDP protocols (Fig. 6B). STDP contrasts SDDP in two main ways: (i) contrarily to SDDP, which elicits bidirectional plasticity for post-pre sequences (sdLTD or sdLTP depending on MSN rheobase) and mainly sdLTD for pre-post sequences, in STDP, the postsynaptic action potential timing determines the strict occurrence of either LTP or LTD and (ii) STDP is induced within a narrower time window (−30<Δt<+30 ms) than SDDP (−110<Δt<+110 ms) (Fig. 6B). Therefore even though the postsynaptic action potential appears determinant for the strict orientation of the plasticity and increases precision of the temporal window of plasticity induction, the present data demonstrates that postsynaptic subthreshold depolarization in coincidence with presynaptic activation is able to induce strong and reliable bidirectional long-term synaptic plasticity.

## Discussion

The striatum, the major input nucleus of the basal ganglia, processes information from the whole cerebral cortex. Due to the specific membrane properties of MSNs, a strong and correlated cortical activity is required to evoke an action potential [21]–[23]. This confers to the striatum the ability to extract relevant information from the background noise and select a cognitive-motor sequence adapted to environmental stimuli [15], [17], [19]. The consequence is that most of cortical activities lead to subthreshold depolarizations in MSNs, as recently reported in awake rats [24] . Therefore, we have tested if brief subthreshold events could be implicated in long-term coding. Some elements suggest that the back-propagating action potential would not be the only postsynaptic depolarizing event necessary for the induction of long-term synaptic plasticity. In the hippocampus, a low-frequency stimulation at 1 Hz induced exclusively LTD whatever the magnitude of postsynaptic depolarization (sub- versus suprathreshold) [12]. Changes of holding membrane potential for relatively long duration (1 minute [9], [10] and 250 ms [11]) paired with theta-burst or action potential, respectively, induced either LTP or LTD, but never bidirectional plasticity. In the cortex, a STDP protocol applied while maintaining the membrane potential at −50 mV induced exclusively LTD, and only LTP at 0 mV [10]. Here, we used a 30 ms subthreshold depolarization to mimic corticostriatal subthreshold summation of EPSPs induced by cortical or thalamic activity (as observed *in vivo* [24]–[26]) paired with a presynaptic cortical stimulation. We observed a robust, reliable and timing-dependent bidirectional long-term plasticity induced by subthreshold events paired with presynaptic cortical activity. Depending on the Δt, we observed the induction of sdLTD or sdLTP for post-pre sequences, depending on the MSN state of excitability, whereas pre-post sequences induced mainly sdLTD. As a postsynaptic depolarization is wider than an action potential, it could be expected that SDDP coding should be temporally rather imprecise. Nevertheless, the occurrence of long-term synaptic efficacy changes induced with SDDP cellular conditioning protocols relies on the exact timing between pre- and postsynaptic activations. Indeed, uncorrelated random paired sequences failed to induce long-term plasticity. Plasticities induced by SDDP sequences are mediated by different receptors. Indeed, sdLTD is mediated by endocannabinoids through CB1 receptor activation and sdLTP is NMDA receptor-activation dependent. Accordingly, sdLTP and sdLTD appear to be mediated by two independent pathways. This pharmacology indicates that brief subthreshold events are very efficiently transmitted throughout the dendritic tree in MSNs. Indeed, in coincidence with a presynaptic activation, a subthreshold depolarization is able to activate NMDA receptors or to induce a synthesis and release of endocannabinoids.

Comparison of corticostriatal SDDP and STDP indicates that a brief postsynaptic subthreshold depolarization is sufficient to induce bidirectional long-term plasticity while a postsynaptic action potential is determinant in the strict orientation of the plasticity and the precision of the time window. As highlighted by STDP, the postsynaptic back-propagating action potential is generally admitted to be the key component for the induction of the long-term plasticity. This means that, besides several types of short- and long-term corticostriatal plasticities such as paired-pulses [30], depolarized-induced suppression of excitation (DSE) [31], [32], Hebbian HFS-LTP [28], [33], non-Hebbian HFS-LTP [28], Hebbian LFS-LTD [28], [34], non-Hebbian LFS-LTD [28] or STDP [28], [35], SDDP extends the capability for MSNs to express long-term plasticity.

Corticostriatal SDDP displayed mainly sdLTD for pre-post sequences and bidirectional plasticity for post-pre sequences. The question was, which parameters were determinant in the orientation of the plasticity induced by post-pre sequences? In pyramidal cells of the cerebral cortex, the localization of synapses on the dendritic tree associated with cable properties of the dendrites is determinant for the orientation (LTP *versus* LTD) of STDP [36], [37] Concerning corticostriatal SDDP, we did not observe any significant correlation between EPSC rise time (an abacus of the electrotonic distance) and the orientation of long-term synaptic efficacy changes. This dissimilarity could be explained by two characteristics of MSNs: (i) all corticostriatal afferents are distributed throughout the spiny part of the dendrites [38], [39] and (ii) a model predicts that the attenuation of corticostriatal EPSPs, generated in the spiny segment of the MSN dendrites, is not tightly related to the electrotonic distance [40]. At corticostriatal synapses, we observed that the occurrence of sdLTD or sdLTP evoked by post-pre SDDP sequence was depending on the MSN rheobase. This finding could be put in relation with the corticostriatal STDP in cholinergic interneurons that shares a similar plasticity orientation [27]; indeed, for both MSN SDDP and cholinergic interneurons STDP, the rheobase was a key parameter in the orientation of plasticity induced by post-pre sequences. Variations in membrane excitability could be due to different intrinsic properties [41], [42], synaptic inputs (glutamatergic, serotoninergic, GABAergic or cholinergic) and/or specific local interactions (chemical and electrical synapses) [43]. Within the corticostriatal network, SDDP and STDP are physiologically relevant for information processing. Indeed, MSNs have been proposed to act as selective coincidence detectors [19], [21]–[23] and this working mode implies that much of cortical activities lead to subthreshold events that nevertheless are able to induce SDDP. Therefore, MSNs have the capability to fully take into account postsynaptic subthreshold signals paired with the cortical activity and, depending on the timing between these activities and neuronal excitability, to generate robust sdLTD or sdLTP. SDDP could have multiple consequences for the corticostriatal transmission. Thus, SDDP and STDP could interact and influence each other for the induction of long-term synaptic efficacy changes at corticostriatal synapses. Indeed, changes in the corticostriatal transmission efficacy induced by SDDP are expected to shift the threshold of MSN coincidence detection and firing. Indeed, LTP induced by theta burst in the hippocampus has been shown to facilitate the coincidence detection [44]. In addition, once the corticostriatal transmission reaches the action potential threshold, a previous induced SDDP should induce a temporal shift of the spike timing and consequently modify the occurrence and magnitude of a subsequent STDP. Such impact of SDDP on STDP is reinforced by the fact that STDP is highly temporally restricted and the temporal position of the action potential has a determinant weight on the induced long-term plasticity orientation and magnitude. Therefore, the occurrence of SDDP could strongly influence the occurrence and magnitude of STDP. In this aspect, SDDP could be seen also as a priming plasticity for STDP. Furthermore, if STDP and SDDP protocols share the same capability to code for bidirectional long-term plasticity, they display, nevertheless, specific properties since STDP is strictly orientated and temporally restricted while SDDP is bidirectional and inducible in a larger time window (−110<Δt<+110 ms *versus* −30<Δt<+30 ms). Therefore, the required conditions for SDDP induction appear much less stringent than those for STDP occurrence. In conclusion, SDDP extends considerably the capabilities of neuronal long-term coding, beyond the action potential.

## Materials and Methods

### Electrophysiological recordings

Patch-clamp recordings of MSNs were performed on horizontal brain slices (330 µm) from Sprague-Dawley rats (postnatal days 15–21). These slices were prepared at the level of the somatosensory cortical area and of the corresponding corticostriatal projection field [27]–[29]. Patch-clamp recordings were made as previously described [27]–[29], [43]. Briefly, borosilicate glass pipettes of 4–7 MΩ resistance contained (mM) for perforated patch-clamp recordings: 105 K-gluconate, 30 KCl, 10 HEPES and 0.3 EGTA (adjusted to pH 7.35 with KOH) and for whole-cell recordings: 105 K-gluconate, 30 KCl, 10 HEPES, 10 phosphocreatine, 4 ATP-Mg, 0.3 GTP-Na, 0.3 EGTA (adjusted to pH 7.35 with KOH). The composition of the extracellular solution was (mM): 125 NaCl, 2.5 KCl, 25 glucose, 25 NaHCO_3_, 1.25 NaH_2_PO_4_, 2 CaCl_2_, 1 MgCl_2_, 10 µM pyruvic acid bubbled with 95% O_2_ and 5% CO_2_. All recordings were performed at 34°C using a temperature control system (Bioptechs ΔTC3, Butler, PA, USA) and slices were continuously superfused at 2–3 ml/min with the extracellular solution. Individual neurons were identified using infrared-differential interference contrast microscopy with CCD camera (Hamamatsu C2400-07; Hamamatsu, Japan). Signals were amplified using an EPC10-2 amplifier (HEKA Elektronik, Lambrecht, Germany). Current-clamp recordings were filtered at 2.5 kHz and sampled at 5 kHz and voltage-clamp recordings were filtered at 5 kHz and sampled at 10 kHz using the program Pulse-8.53 (HEKA Elektronik). The series resistance was compensated at 75–80%.

All electrophysiological recordings were realized in perforated patch-clamp configuration (except experiments with biocytin or BAPTA that were performed in whole-cell configuration) at 32°C and without any pharmacological treatments or ionic modifications to preserve the local striatal microcircuits involved in corticostriatal transmission. All chemicals were purchased by Sigma (Saint Quentin, France) except 6-cyano-7- nitroquinoxaline-2,3-dione (CNQX), DL-2-Amino-5-phosphonopentanoic acid (AP5) and AM251 (Tocris, Ellisville, MO, USA).

### Perforated patch-clamp recordings

Amphotericin B (Sigma-Aldrich) was used to perform perforated patch-clamp experiments, as previously described [27], [29]. The concentration of amphotericin B in the patch-clamp pipette solution was 200 µg/ml. Perforated patch prevents a dialysis of intracellular content and therefore avoids a loss of intracellular molecules that could be involved in long-term plasticity occurrence and maintenance.

### Biocytin filling and histochemistry

Biocytin (Sigma) 5 mg/ml was dissolved into the patch-clamp pipette solution and cells were filled during at least 20 min of recording (performed at 34°C). Subsequently, slices were fixed overnight in 2% paraformaldehyde at 4°C. Biocytin-filled cells were visualized using the avidin-biotin-horseradish peroxidase reaction (ABC Elite peroxidase kit; Vector Laboratories, Burlingame, CA, USA) according to the instructions of the manufacturer, or streptavidin-alexa488 (Invitrogen, Carlsbad, CA, USA), incubated 2 h at room temperature.

### Stimulation protocols

Electrical stimulations of the cerebral cortex were performed with a bipolar electrode (Phymep, Paris, France) placed in the layer V of the somatosensory cortex [27]–[29]. Electrical stimulations were monophasic at constant current (Stimulator WPI, Stevenage, UK), without detectable polarization of electrodes along time. There was no significant difference in the current intensities of cortical stimulations between each stimulation protocol group: post-pre and pre-post SDDP sequences. This indicates that the orientation of induced synaptic plasticities (LTP *versus* LTD) was not related to the stimulation intensity. Currents were adjusted in order to evoke striatal EPSCs ranging from 50 to 200 pA amplitudes. Repetitive control stimuli were applied at 0.1 Hz, a frequency for which neither short- nor long-term synaptic efficacy changes in EPSC amplitudes were induced [28].

SDDP experiments consisted in pairing presynaptic stimulus and MSN postsynaptic brief subthreshold depolarization (30 ms duration) at a defined time interval, which was varied between experiments. Concerning uncorrelated SDDP experiments, the time interval of pre- and postsynaptic pairing was randomly varied for each sweep of the pairing period (−50<Δt_random_<0 ms or 0<Δt_random_<+50 ms). STDP experiments consisted of time shifting the presynaptic stimulation with a postsynaptic action potential evoked by a direct application of a depolarizing current step (30 ms duration). Cortical stimulations and evoked subthreshold depolarizations (correlated or uncorrelated SDDP) or action potentials (STDP) in MSNs were both delivered 100 times at 1 Hz. Neurons were recorded for 10 minutes in control and for at least one hour after the cellular conditioning protocol; long-term synaptic efficacy changes were measured around one hour. Input resistance was monitored throughout the experiments and a variation superior to 20% led to the rejection of the experiment. Drugs were applied in the bath (except BAPTA, applied intracellularly), after recording 10 minutes of baseline and 10 minutes before cellular conditioning protocol, and were present continuously until the end of the recording.

### Data analysis

Off-line analysis was performed using Igor-Pro (Wavemetrics, Lake Oswego, OR, USA) and Prism (GraphPad, La Jolla, CA, USA). All results were expressed as mean±SEM (except in Fig. 6B, in which Box and Whiskers plots were represented) and statistical significance was assessed using the Student's t test, the non-parametric Mann-Whitney test when appropriate or the Pearson test for correlations at the significance level (p) indicated. EPSC mean amplitudes, measured 60 minutes after induction protocol, were the average of 26 evoked EPSCs (each 26 EPSC was normalized to the mean of EPSC amplitudes recorded before induction protocol). Synaptic efficacy changes were classified as either LTP or LTD when the mean of normalized EPSCs amplitudes was significantly different from the control baseline (Fig. 2B, inserts).

Plasticity loci were determined by the mean variance analysis method [45]. Briefly, EPSC coefficients of variation (CV) were calculated by the ratio of the standard deviation and the mean EPSC amplitude. The plasticity loci were deduced from the relationship between the normalized CV^−2^ (CV^−2^ after induction of plasticity/CV^−2^ control) and the normalized EPSC amplitudes (EPSC mean amplitude after induction of plasticity/control EPSC mean amplitude). EPSC amplitude is proportional to *npq* with *n* being the number of releasing sites, *p* the probability of release and *q* the quantum size. It is assumed that *n* and *p* describe presynaptic events and *q* is a post-synaptic indicator. If normalized CV^−2^>normalized EPSC amplitude, both *n* and *p* can vary, and if normalized CV^−2^ = normalized EPSC amplitude, only *n* can vary. In both cases, changes in EPSC amplitude reflect mainly presynaptic modifications. If normalized CV^−2^<normalized EPSC amplitude, changes in EPSC amplitude are related to variations of *n*, *p* and *q*, indicating a mixed (pre- and postsynaptic) origin of the modifications. Finally, a variation of normalized EPSC amplitude without any variation of normalized CV^−2^ indicates postsynaptic modifications since only *q* can vary.

## Supporting Information

Figure S1 Characterization of SDDP. The magnitudes of long-term synaptic efficacy changes were plotted against EPSC rise time in control (A), EPSC latency in control (B), EPSC amplitude in control (C), RMP (D) and input resistance (E). No significant correlation was found between these parameters and the magnitude of long-term synaptic efficacy changes induced by SDDP protocols (r values are indicated in each graph).
(0.59 MB TIF)

Figure S2 Pharmacology of plasticity induced by post-pre and pre-post sequences. (A) For post-pre sequences (−50≤Δt≤0 ms), with AP5, sdLTP was no longer observed while sdLTD could still be induced (−14.7±10.3%, n = 6). Conversely, with AM251, we did not observe significant sdLTD while sdLTP still occurred (+15.5±14.3%, n = 5). (B) For pre-post sequences (0≤Δt≤+50 ms) similar results were observed. Indeed, with AP5, sdLTD was mainly induced (−13.4±13%, n = 6) whereas, with AM251, we observed either sdLTP or no plasticity (+19.3±11.4%, n = 5).
(1.29 MB TIF)

## References

[1] Lynch MA (2004). Long-term potentiation and memory.. Physiol Rev.

[2] Martin SJ, Grimwood PD, Morris RG (2000). Synaptic plasticity and memory: an evaluation of the hypothesis.. Annu Rev Neurosci.

[3] Martin SJ, Morris RG (2002). New life in an old idea: the synaptic plasticity and memory hypothesis revisited.. Hippocampus.

[4] Abbott LF, Nelson SB (2000). Synaptic plasticity: taming the beast.. Nat Neurosci.

[5] Bi G, Poo M (2001). Synaptic modification by correlated activity: Hebb's postulate revisited.. Annu Rev Neurosci.

[6] Dan Y, Poo MM (2004). Spike timing-dependent plasticity of neural circuits.. Neuron.

[7] Dan Y, Poo MM (2006). Spike timing-dependent plasticity: from synapse to perception.. Physiol Rev.

[8] Sjostrom PJ, Nelson SB (2002). Spike timing, calcium signals and synaptic plasticity.. Curr Opin Neurobiol.

[9] Artola A, Brocher S, Singer W (1990). Different voltage-dependent thresholds for inducing long-term depression and long-term potentiation in slices of rat visual cortex.. Nature.

[10] Feldman DE (2000). Timing-based LTP and LTD at vertical inputs to layer II/III pyramidal cells in rat barrel cortex.. Neuron.

[11] Sjostrom PJ, Turrigiano GG, Nelson SB (2004). Endocannabinoid-dependent neocortical layer-5 LTD in the absence of postsynaptic spiking.. J Neurophysiol.

[12] Staubli UV, Ji ZX (1996). The induction of homo- vs. heterosynaptic LTD in area CA1 of hippocampal slices from adult rats.. Brain Res.

[13] Graybiel AM (1995). Building action repertoires: memory and learning functions of the basal ganglia.. Curr Opin Neurobiol.

[14] Graybiel AM (2005). The basal ganglia: learning new tricks and loving it.. Curr Opin Neurobiol.

[15] Houk JC, Wise SP (1995). Distributed modular architectures linking basal ganglia, cerebellum, and cerebral cortex: their role in planning and controlling action.. Cereb Cortex.

[16] Packard MG, Knowlton BJ (2002). Learning and memory functions of the Basal Ganglia.. Annu Rev Neurosci.

[17] Redgrave P, Prescott TJ, Gurney K (1999). The basal ganglia: a vertebrate solution to the selection problem?. Neuroscience.

[18] Yin HH, Knowlton BJ (2006). The role of the basal ganglia in habit formation.. Nat Rev Neurosci.

[19] Graybiel AM, Aosaki T, Flaherty AW, Kimura M (1994). The basal ganglia and adaptive motor control.. Science.

[20] Wilson CJ (1995). The contribution of cortical neurons to the firing pattern of striatal spiny neurons..

[21] Calabresi P, Misgeld U, Dodt HU (1987). Intrinsic membrane properties of neostriatal neurons can account for their low level of spontaneous activity.. Neuroscience.

[22] Nisenbaum ES, Wilson CJ (1995). Potassium currents responsible for inward and outward rectification in rat neostriatal spiny projection neurons.. J Neurosci.

[23] Nisenbaum ES, Xu ZC, Wilson CJ (1994). Contribution of a slowly inactivating potassium current to the transition to firing of neostriatal spiny projection neurons.. J Neurophysiol.

[24] Mahon S, Vautrelle N, Pezard L, Slaght SJ, Deniau JM, Chouvet G, Charpier S (2006). Distinct patterns of striatal medium spiny neuron activity during the natural sleep-wake cycle.. J Neurosci.

[25] Stern EA, Jaeger D, Wilson CJ (1998). Membrane potential synchrony of simultaneously recorded striatal spiny neurons in vivo.. Nature.

[26] Stern EA, Kincaid AE, Wilson CJ (1997). Spontaneous subthreshold membrane potential fluctuations and action potential variability of rat corticostriatal and striatal neurons in vivo.. J Neurophysiol.

[27] Fino E, Deniau JM, Venance L (2008). Cell-specific spike-timing-dependent plasticity in GABAergic and cholinergic interneurons in corticostriatal rat brain slices.. J Physiol.

[28] Fino E, Glowinski J, Venance L (2005). Bidirectional activity-dependent plasticity at corticostriatal synapses.. J Neurosci.

[29] Fino E, Paille V, Deniau JM, Venance L (2009). Asymmetric spike-timing dependent plasticity of striatal nitric oxide-synthase interneurons.. Neuroscience.

[30] Fitzpatrick JS, Akopian G, Walsh JP (2001). Short-term plasticity at inhibitory synapses in rat striatum and its effects on striatal output.. J Neurophysiol.

[31] Narushima M, Hashimoto K, Kano M (2006). Endocannabinoid-mediated short-term suppression of excitatory synaptic transmission to medium spiny neurons in the striatum.. Neurosci Res.

[32] Uchigashima M, Narushima M, Fukaya M, Katona I, Kano M, Watanabe M (2007). Subcellular arrangement of molecules for 2-arachidonoyl-glycerol-mediated retrograde signaling and its physiological contribution to synaptic modulation in the striatum.. J Neurosci.

[33] Charpier S, Deniau JM (1997). In vivo activity-dependent plasticity at cortico-striatal connections: evidence for physiological long-term potentiation.. Proc Natl Acad Sci U S A.

[34] Calabresi P, Maj R, Pisani A, Mercuri NB, Bernardi G (1992). Long-term synaptic depression in the striatum: physiological and pharmacological characterization.. J Neurosci.

[35] Pawlak V, Kerr JN (2008). Dopamine receptor activation is required for corticostriatal spike-timing-dependent plasticity.. J Neurosci.

[36] Letzkus JJ, Kampa BM, Stuart GJ (2006). Learning rules for spike timing-dependent plasticity depend on dendritic synapse location.. J Neurosci.

[37] Sjostrom PJ, Hausser M (2006). A cooperative switch determines the sign of synaptic plasticity in distal dendrites of neocortical pyramidal neurons.. Neuron.

[38] Chang HT, Wilson CJ, Kitai ST (1982). A Golgi study of rat neostriatal neurons: light microscopic analysis.. J Comp Neurol.

[39] Smith AD, Bolam JP (1990). The neural network of the basal ganglia as revealed by the study of synaptic connections of identified neurones.. Trends Neurosci.

[40] Wilson CJ (1984). Passive cable properties of dendritic spines and spiny neurons.. J Neurosci.

[41] Fino E, Glowinski J, Venance L (2007). Effects of acute dopamine depletion on the electrophysiological properties of striatal neurons.. Neurosci Res.

[42] Venance L, Glowinski J (2003). Heterogeneity of spike frequency adaptation among medium spiny neurones from the rat striatum.. Neuroscience.

[43] Venance L, Glowinski J, Giaume C (2004). Electrical and chemical transmission between striatal GABAergic output neurones in rat brain slices.. J Physiol.

[44] Xu NL, Ye CQ, Poo MM, Zhang XH (2006). Coincidence detection of synaptic inputs is facilitated at the distal dendrites after long-term potentiation induction.. J Neurosci.

[45] Clements JD, Silver RA (2000). Unveiling synaptic plasticity: a new graphical and analytical approach.. Trends Neurosci.

