# Supplementary figures and images for: Brief Subthreshold Events Can Act as Hebbian Signals for Long-Term Plasticity
Source: PLoS One. 2009 Aug 7;4(8):e6557. doi: 10.1371/journal.pone.0006557 (PMC2725411; doi:10.1371/journal.pone.0006557)

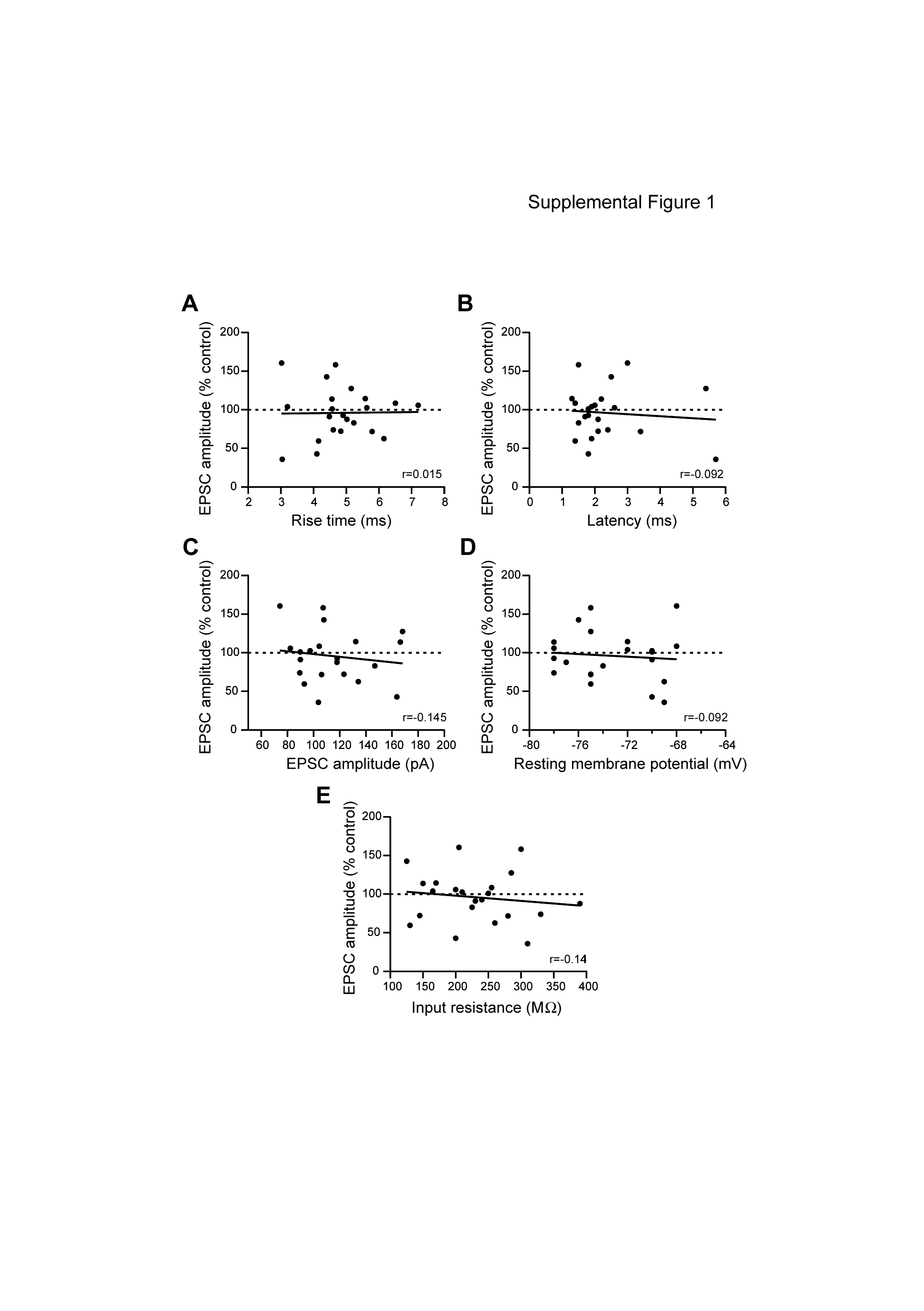

Supplement: Figure S1 — Characterization of SDDP. The magnitudes of long-term synaptic efficacy changes were plotted against EPSC rise time in control (A), EPSC latency in control (B), EPSC amplitude in control (C), RMP (D) and input resistance (E). No significant correlation was found between these parameters and the magnitude of long-term synaptic efficacy changes induced by SDDP protocols (r values are indicated in each graph). (0.59 MB TIF) [file pone.0006557.s001.tif]

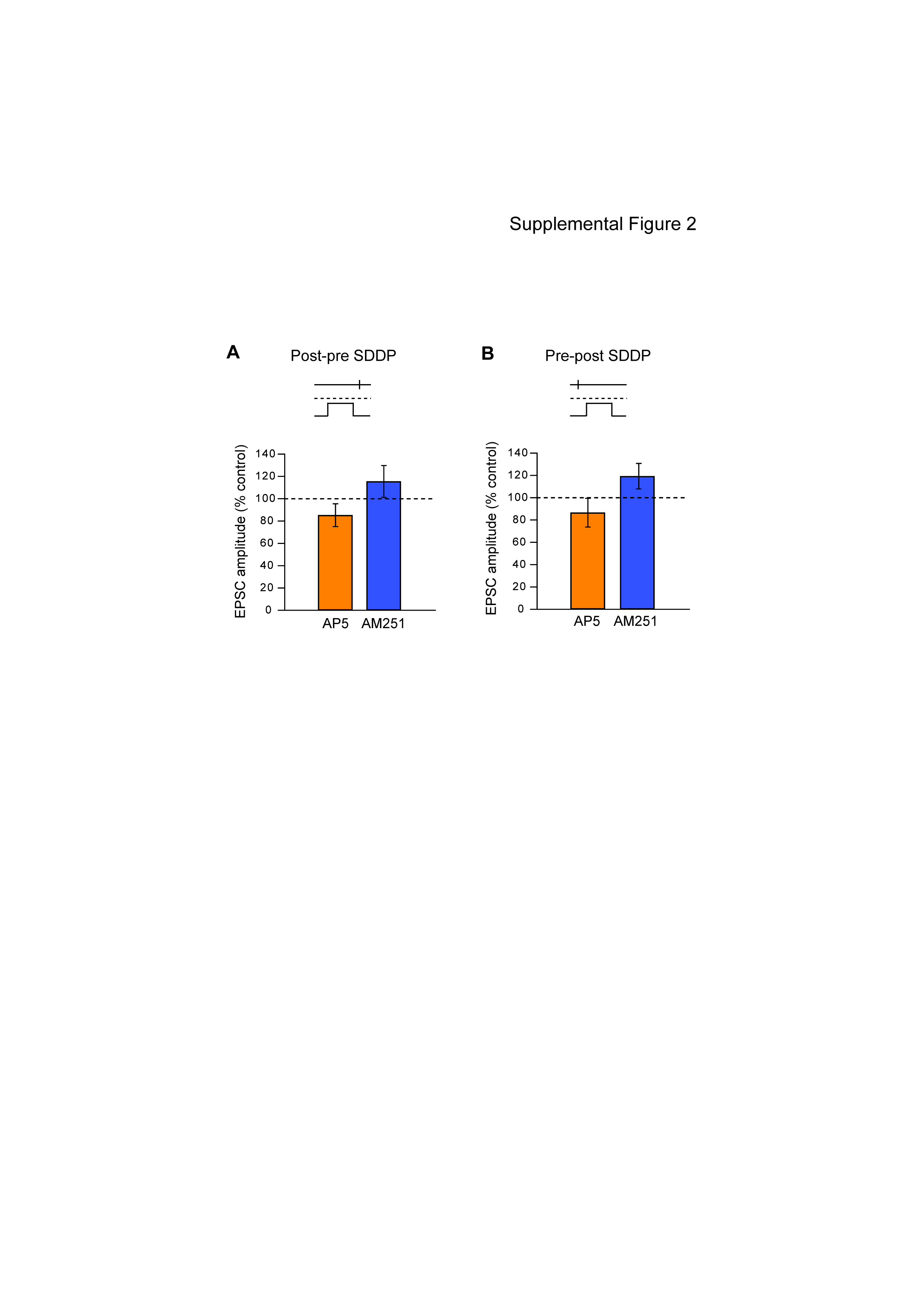

Supplement: Figure S2 — Pharmacology of plasticity induced by post-pre and pre-post sequences. (A) For post-pre sequences (−50≤Δt≤0 ms), with AP5, sdLTP was no longer observed while sdLTD could still be induced (−14.7±10.3%, n = 6). Conversely, with AM251, we did not observe significant sdLTD while sdLTP still occurred (+15.5±14.3%, n = 5). (B) For pre-post sequences (0≤Δt≤+50 ms) similar results were observed. Indeed, with AP5, sdLTD was mainly induced (−13.4±13%, n = 6) whereas, with AM251, we observed either sdLTP or no plasticity (+19.3±11.4%, n = 5). (1.29 MB TIF) [file pone.0006557.s002.tif]
